# Accuracy of the Electrocardiogram in Localizing the Accessory Pathway in Patients with Wolff-Parkinson-White Pattern

**DOI:** 10.5935/abc.20160132

**Published:** 2016-10

**Authors:** Carlos Manuel Teixeira, Telmo António Pereira, Ana Margarida Lebreiro, Sérgio Alexandre Carvalho

**Affiliations:** 1 Instituto Politécnico de Coimbra - ESTESC - Departamento de Fisiologia Clínica Coimbra - Portugal; 2 Centro Hospitalar São João - Serviço de Cardiologia Porto - Portugal

**Keywords:** Electrocardiography, Wolff-Parkinson-White Syndrome, Catheter Ablation, Accessory Atrioventricular Bundle, Data Accuracy

## Abstract

**Background:**

There are currently several electrocardiographic algorithms to locate the accessory pathway (AP) in patients with Wolff-Parkinson-White (WPW) syndrome.

**Objective:**

To compare the ability of electrocardiographic algorithms in identifying the location of the AP in patients with WPW pattern referred for ablation.

**Methods:**

Observational, cross-sectional, retrospective study with 111 patients with WPW syndrome referred for AP ablation. The electrocardiogram (ECG) obtained prior to the ablation was analyzed by an experienced observer who consecutively applied seven algorithms to identify non-invasively the AP. We then compared the location estimated with this assessment with that obtained in the electrophysiological study and calculated the agreement rates.

**Results:**

Among the APs, 59 (53.15%) were distributed around the mitral annulus and the remaining 52 (46.85%) were located around the tricuspid annulus. The overall absolute accuracy of the algorithms evaluated varied between 27% and 47%, increasing to between 40% and 76% when we included adjacent locations. The absolute agreement rate by AP location was 2.00-52.20% for septal APs (n = 51), increasing to 5.90-90.20% when considering adjacent locations; 7.70-69.20% for right APs (n = 13), increasing to 42.90-100% when considering adjacent locations; and 21.70-54.50% for left APs (n = 47), increasing to 50-87% when considering adjacent locations.

**Conclusion:**

The agreement rates observed for the analyzed scores indicated a low discriminative ability of the ECG in locating the AP in patients with WPW.

## Introduction

In 1930, Wolff, Parkinson, and White described a syndrome, later named after them, that affected young patients without structural heart disease, manifesting with a short PR interval, wide QRS complex, and episodes of paroxysmal tachycardia in the electrocardiogram (ECG).^1,2^

The Wolff-Parkinson-White syndrome (WPW) is a form of ventricular preexcitation in which part of the ventricular myocardium is depolarized early by one or more accessory pathways (APs) that bypass the atrioventricular (AV) node, establishing a direct link between the atrium and the ventricle.^3,4^

The APs result from an abnormal embryological development of the myocardium during differentiation of the fibrous tissue that separates the atria and ventricles. They are classified based on their location, number, direction, and conduction properties.^5-7^

There are currently two basic therapeutic options for patients with WPW: pharmacological therapy and catheter ablation. Radiofrequency catheter ablation is a safe, effective, and curative approach, given its high individual effectiveness.^3,8^ The approach used for the electrophysiologic study (EPS) and ablation depends on the location of the AP, which should, whenever possible, be established by ECG. Prior knowledge of the AP location allows better planning, faster and safer procedure, as well as decreased exposure to ionizing radiation and unnecessary punctures, allowing an early choice of appropriate catheters and energy sources.^9,10^

Since the introduction of ablation, several algorithms to predict the AP location have been published.^11-17^ Each algorithm considers distinct electrocardiographic criteria, has different techniques and "gold standards," adopts different nomenclatures and number of identified regions, and presents decreased discriminative ability in the presence of multiple APs, myocardial infarction, and left ventricular hypertrophy. In preliminary results, the algorithms showed good discriminating ability and their use should be considered as a guide to locate the AP.^9,10^

The aim of this study was focused on the comparative evaluation of the discriminative ability of electrocardiographic scores in locating the AP in patients with WPW syndrome referred for radiofrequency catheter ablation, seeking to identify the best algorithm currently available for use in clinical practice.

## Methods

### Study design and sample selection

This was an observational, cross-sectional, and retrospective study based on the analysis of the electrocardiographic parameters of WPW, sequential use of electrocardiographic algorithms, and location of the AP in the EPS.

After approval by the Administrative Committee and Ethics Committee of *Centro Hospitalar S. João - EPE*, and compliance with ethical criteria such as anonymity, confidentiality, and use of the collected data only for scientific purposes, we conducted a survey of ECGs and results of EPSs performed by 111 patients with a WPW pattern.The study included a nonprobabilistic, convenience cohort identified from an entire sample of patients available in the clinical files of the Cardiology Department at *Centro Hospitalar S. João - EPE*, between June 1, 2007, and December 31, 2012.

The inclusion criteria were: (1) an ECG prior to the EPS/ablation, showing the WPW pattern in sinus rhythm; (2) EPS/ablation indicating the location of the AP; (3) a single AP; and (4) a structurally normal heart. We excluded those individuals presenting with the following: (1) an ECG with a pattern of intermittent WPW; (2) diagnosis of multiple APs; (3) diagnosis of structural heart disease; (4) ECG performed after the EPS/ablation; (5) lack of information about the location of the AP in the EPS.

### Electrocardiographic algorithms

We analyzed seven electrocardiographic algorithms that locate noninvasively the AP in patients with WPW pattern, proposed by Arruda et al.,^11^ Boersma et al.,^12^ Chiang et al.,^13^ D'Avila et al.,^14^ Fitzpatrick et al.,^15^ Iturralde et al.,^16^ and Xie et al.^17^ The seven algorithms divide the AP annulus into 5-13 regions, using diverse combinations of criteria for analysis, namely, polarity of the QRS complex, amplitude and duration of the R wave, and amplitude and polarity of the delta wave. To accurately compare the algorithms, we used the 13 locations described by Chiang et al. following the analogy proposed by Wren et al.^9^ (Table 1).

**Table 1:** Comparison between the number of AP locations and the terminology used in each algorithm

	Arruda	Boersma	Chiang	D'Avila	Fitzpatrick	Iturralde	Xie
Locations	13	7	13	8	8	5	9
LAL	LAL	LL	LAL	LL	LAL	LPL/LAS	LAL
LL	LL	LL	LL	LL	LAL	LPL/LAS	LPL
LPL	LPL	LPS	LPL	LP	LPL	LIP/LI	LPL
LP	LP	LPS	LP	LParaS	LPL	LIP/LI	LP
LPS	PSMA	LPS	LPS	LParaS	LPS	LIP/LI	LPS
RPS	PSTA	PS	RPS	PS	RPS	RIP/RI	RPS
RMS	MSTA	MS	RMS	MS	RMS	RASP	MS
RAS	AS/RAPS	MS	RAS	MS	RAS	RASP	RAS
RA	RA	AS	RA	AS	RAL	RA	RA/RL
RAL	RAL	RL	RAL	RL	RAL	RA	RA/RL
RL	RL	RL	RL	RL	RAL	RA	RA/RL
RPL	RPL	RL	RPL	RParaS	RPL	RIP/RI	RA/RL
RP	RP	RPS	RP	RParaS	RPL	RIP/RI	RP

The locations indicate the number of possible locations with each algorithm. LAL: left anterolateral; RASP: right anterosuperior paraseptal; LL: left lateral; LPL: left posterolateral; LAS: left anterosuperior; LP: left posterior; LPS: left posteroseptal; RPS: right posteroseptal; RMS: right midseptal; RAS: right anteroseptal; RA: right anterior; RAL: right anterolateral; RL: right lateral; RPL: right posterolateral; RP: right posterior; PSMA: posteroseptal mitral annulus; PSTA: posteroseptal tricuspid annulus; MSTA: midseptal tricuspid annulus; AS: anteroseptal; AS/RAPS: anteroseptal / right anterior paraseptal; PS: posteroseptal; MS: midseptal; LParaS: left paraseptal; RParaS: right paraseptal; LIP / LI: left inferior paraseptal / left inferior; RIP / RI: right inferior paraseptal / right inferior.

### Procedure

A member of the research team trained in the application of the evaluated algorithms and with expertise in ECG analyzed all 111 ECGs available in our sample. Simultaneously, this researcher distributed blindly and independently 10 random copies of 10 different ECGs from the sample with the help of two other members of the team, instructing each researcher to identify the AP using the same methodology.

The locations obtained with the consecutive application of the seven algorithms were organized according to possible locations (between 1-13), as shown in Table 1. The locations were then compared with those determined by the EPS during radiofrequency ablation. For each algorithm, we established the following: (1) accurate - the location of the AP in the EPS matched that predicted by the algorithm; (2) adjacent location - the location of the AP predicted by the algorithm was anatomically adjacent to the location determined in the EPS; (3) inaccurate - the location of the AP predicted by the algorithm and determined by the EPS were in different anatomic positions.

Regarding the 10 ECGs analyzed by different members of the research team, we established the following: (1) agreement among observers - similar AP locations in each algorithm; (2) disagreement among researchers - differences in the results of the application of each algorithm, regardless of the agreement with the results observed in the EPS.

### Statistical analysis

After data collection and summary, we performed statistical analyses using the software SPSS, version 19.0 (IBM SPSS Statistics for Windows).

We initially conducted a simple descriptive statistical analysis, calculating the mean values ± standard deviations, and absolute and relative frequencies to characterize the sample's variables.

To verify the normal distribution of the continuous variables, we used the Kolmogorov-Smirnov test. We used parametric statistical tests for data with normal distribution and nonparametric statistical tests for those without a normal distribution. To compare continuous variables between both groups, we used Student's *t* test for independent samples or the Mann-Whitney U test, as appropriate. For comparisons of categorical variables, we used the chi-square test. Instead, when the number of cases in any cell of the contingency table was below five, we used Fisher's exact test.

To study the overall accuracy of the algorithms, we used the statistical approach proposed by Wren et al.^9^ Considering that the algorithms identify a different number of anatomical regions around the AV annulus, the hypothesis to predict the AP location successfully (accuracy) is proportional to the number of regions for each algorithm. Thus, an algorithm with eight locations will have a probable accuracy of 1:8 for each attempt. We used a ratio test to compare the accuracy rates for each algorithm, taking into account the obtained number of anatomical locations and number of observations. The agreements observed were compared with the predictions for each attempt, according to the aforementioned probability ratio. The success obtained (p) was subtracted by the probability for each trial (p_e_), and the result was divided by the standard error of the mean (SEM). For example, for an algorithm with 13 possible locations and an obtained agreement of 40%, p = 0.4 and p_e_ = 0.08 (*i.e.*, 1/13).

We also evaluated the agreement rate between the observers by comparing the results of the assessment of each algorithm by the members of the research team in the 10 random ECGs.

The interpretation of the statistical tests was based on the significance level α = 0.05 with a 95% confidence interval.

## Results

The sample comprised 111 patients with a mean age of 36.54 ± 15.26 years, including 67 males and 44 females. There were no statistically significant differences between the average age of each sex (males = 34.61 ± 14.87 years, females = 39.48 ± 15.55 years). Most subjects (77.20%) were symptomatic, and palpitation was the most frequently reported symptom among individuals of both sexes.

During the EPS, 49 patients (47.10%) developed tachyarrhythmias, including orthodromic atrioventricular nodal reentrant tachycardia (AVNRT), which was the most common arrhythmia in both sexes (24 subjects), followed by atrial fibrillation (14 individuals, 13.50%), and antidromic AVNRT (two individuals, 1.90%). In one individual, the cardiac rhythm changed from atrial fibrillation to ventricular fibrillation during the procedure. We observed 31 patients (27.90%) with an AP refractory period ≤ 240 ms, which has been recognized as a risk marker of sudden death in patients with WPW syndrome.^18,19^ The APs evaluated in the study showed an ability to conduct the stimulus in both anterograde and retrograde directions in most individuals of both sexes (60 men [54.10%] and 39 women [35.10%]), with no significant differences between sexes (p > 0.05 ). The EPS also revealed that only eight individuals (7.20%) had an intermittent WPW pattern, with no significant differences in the type of WPW pattern by sex (p > 0.05).

The EPS revealed that most APs were located in the posteroseptal right area (28 individuals, 25.20%) and left lateral area (27 individuals, 24.30%). The right anterolateral area was the least represented in the sample (one individual). Overall, 59 APs (53.15%) were distributed around the mitral annulus, while the remaining 52 APs (46.85%) were distributed around the tricuspid annulus. We found no statistically significant differences between sexes in the distribution of the APs around the AV annuluses (p > 0.05).

**Figure 1:**
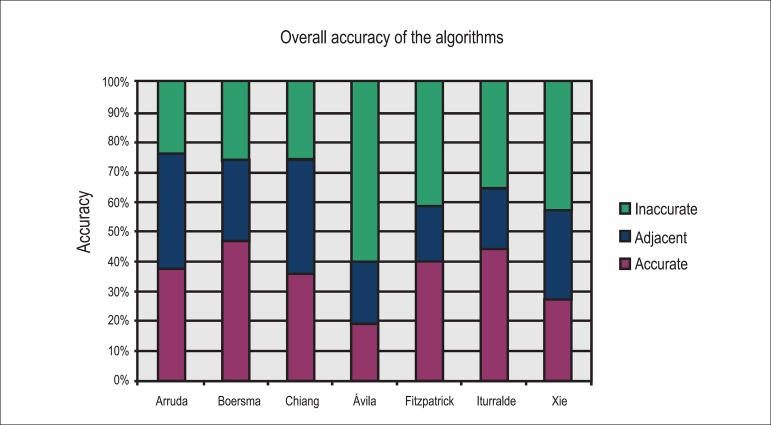
Overall accuracy of the seven analyzed algorithms.

Figure 1 and Table 2 show the overall accuracy of the algorithms.

**Table 2:** Accuracy rates for each analyzed algorithm (n = 111)

	Algorithms analyzed						
	Arruda	Boersma	Chiang	D'Avila	Fitzpatrick	Iturralde	Xie
Locations	13	7	13	8	8	5	9
Accurate (%)	37.80	47.20	36.00	19.80	39.60	44.10	27.00
Adjusted accurate	4.07	2.86	3.83	0.96	2.17	2.87	2.04
Accurate + Adjacent	75.60	73.60	74.70	39.60	58.50	64.80	56.70

With the application of the algorithms analyzed in the study, we observed agreement rates ranging from 19.80% to 47.20% for the exact AP location as the one determined in the EPS. This value increased when we accepted all adjacent locations, ranging from 39.60% (algorithm by D'Avila et al.^14^) to 75.60% (algorithm by Arruda et al.^11^). The average value of "no agreement" for the seven algorithms including all anatomical locations was 36.64%. The accuracy rate corrected for the number of possible anatomic locations was greater with the algorithm by Arruda et al.^11^ (about 4.07 more matches relative to the number of possible matches per location, in the probability per chance expected for this algorithm), and the algorithm by Chiang et al.^13^ (3.83 times more matches).

The agreement rates of the results obtained by the research team members (Table 3) ranged between 40% and 80%. The algorithm by D'Avila et al.^14^ (eight possible locations) and Iturralde et al.^16^ (five possible locations) emerged as those with highest agreement rates: 80% and 70%, respectively. The algorithms by Arruda et al.,^11^ Chiang et al.,^13^ and Fitzpatrick et al.^15^ had the lowest agreement rates (40% for Arruda et al.^11^ and 50% for Chiang et al.^13^ and Fitzpatrick et al.^15^). Considering all seven algorithms, we found that the average agreement rate was 58.57%. This value increased to 64% with algorithms that identify five to nine locations and decreased to 45% with those that identify 13 AP locations.

**Table 3:** Agreement rates for each analyzed algorithm (n = 10)

	Algorithms analyzed						
	Arruda	Boersma	Chiang	D'Avila	Fitzpatrick	Iturralde	Xie
Locations	13	7	13	8	8	5	9
Agreement (among observers - %)	40.00	60.00	50.00	80.00	50.00	70.00	60.00

**Figure 2:**
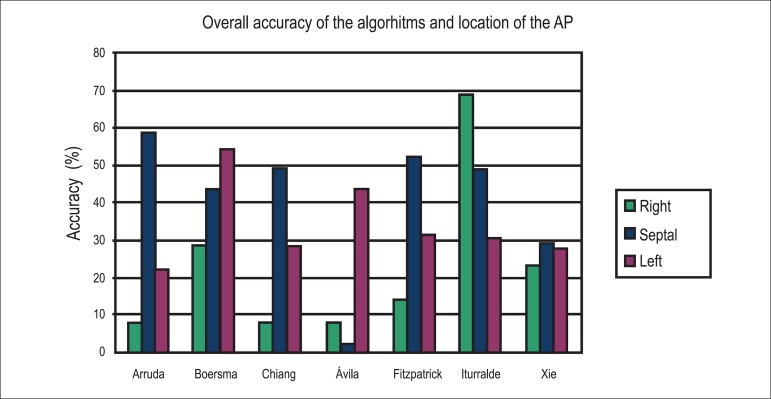
Graph display of the accuracy of the seven algorithms for APs with right, septal and left locations.

Figure 2 and Table 4 show the overall accuracy of the algorithms for septal, right, and left APs, observed in 51, 13, and 47 individuals, respectively.

**Table 4:** Accuracy rates calculated with the seven algorithms for APs with right, septal, and left locations

						Algorithms analyzed			
			Arruda	Boersma	Chiang	D'Avila	Fitzpatrick	Iturralde	Xie
		Locations	13	7	13	8	8	5	9
Accessory pathway	Septal (n= 51)	Accurate (%)	58.80	43.50	49.00	2.00	52.20	49.00	29.40
		Accurate + Adjacent	90.20	50.50	78.40	5.90	69.60	68.60	52.90
	Right (n= 13)	Accurate (%)	7.70	28.60	7.70	7.70	14.30	69.20	23.10
		Accurate + Adjacent	69.20	100	61.50	100	42.90	84.60	53.90
	Left (n= 47)	Accurate (%)	21.70	54.50	28.30	43.50	31.80	30.40	27.90
		Accurate + Adjacent	60.80	63.60	74.00	87.00	50.00	54.30	64.90

For septal APs, the accuracy of the location (match) ranged between 2.00% and 58.80% (Arruda et al.^11^) and increased to 5.90-90.20% when adjacent locations were considered. The average accuracy of all algorithms was 40.56%, with the algorithm by D'Avila et al.^14^ showing a significantly lower agreement rate than the average. Excluding the algorithms by D'Avila et al.^14^ and Xie et al.,^17^ the agreement rates were similar for any algorithm that identified between five and 13 AP locations.

For the right APs, the agreement rate varied between 7.70% and 69.20%, increasing to 42.90% to 100% when adjacent locations were considered. The algorithm by Iturralde et al.^16^ had the highest agreement rate (69.20%) compared to the average (22.61%). Excluding the algorithm by D'Avila et al.,^14^ the algorithms that identified 13 locations obtained agreement rates more distant from the average.

In the left APs, the success rate varied between 21.70% and 54.50%, increasing to 50% to 87% when adjacent locations were included. The algorithm by Boersma et al.^12^ had the highest agreement rate (54.50%) relative to the average (33.44%).

For the results presented in Table 4, we observed no significant differences regarding sex and location of septal, right, and left APs for each algorithm (p > 0.05).

## Discussion

In this study, we tested seven different algorithms potentially locating the AP in 5-13 possible positions, proposed by Arruda et al.,^11^ Boersma et al.,^12^ Chiang et al.,^13^ D'Avila et al.,^14^ Fitzpatrick et al.,^15^ Iturralde et al.,^16^ and Xie et al.^17^ The objective was to evaluate the diagnostic capability of the 12-lead ECG in locating the AP in patients with a WPW pattern referred to EPS and radiofrequency catheter ablation. The sample consisted of 111 individuals, 67 males (60.36%) and 44 females (39.64%), aged between 7 and 75 years (mean 36.54 ± 15.27 years). We found a significant difference between the number of men and women with a WPW pattern. This finding is in agreement with the results published by Cain et al.^20^ who found a similar prevalence (60.90%) in men diagnosed with the WPW syndrome.

We observed that 47.10% of the patients presented arrhythmias during the EPS, which is in line with findings by Brembilla-Perrot et al.,^21^ who reported that approximately 50% of the individuals with a WPW pattern develop tachyarrhythmia. In our study, the most common arrhythmia was AVNRT, including orthodromic AVNRT, which was common in both sexes, as expected.^22^

A retrospective comparison of the ability of the seven analyzed algorithms in identifying a single AP in 111 individuals revealed a precise location for the AP in only 27-47.20% of the cases. The algorithms by Arruda et al.^11^ and Chiang et al.^13^ had the highest agreement rates when corrected for the number of possible anatomical locations (4.07 and 3.83 times higher, respectively). These results show that the prediction of a precise AP location is not directly related to the number of possible locations identified by each algorithm, since even when adjacent locations are considered as correct, the agreement rate for each individual algorithm approached the overall average agreement rate (63.36%) of the algorithms evaluated in the study. However, the agreement between the researchers (regardless of accuracy) seems to have been influenced by the number of possible locations for each algorithm since the agreement rates were superior for algorithms identifying fewer possible locations (five to nine locations, average agreement of 64%).

The agreement rates for septal APs (between 2.00% and 52.20%) were far from those expected. However, the inclusion of adjacent locations showed that the prediction of the approximate location with the algorithm by Arruda et al.^11^ (with 13 locations) had a rate similar to that expected (90.20%). Thus, this was the most appropriate algorithm to estimate the approximate location of septal APs. For right APs, which were present in 13 individuals, we found an agreement rate of 7.70-69.20%. Although these values were far from those expected, the algorithm by Iturralde et al. (five locations) showed the best results in locating the AP correctly. The inclusion of adjacent APs revealed that the algorithm by Boersma et al.^12^ (seven locations) was the most suitable to identify the approximate location of right APs (100% accuracy). Finally, the agreement rates that we obtained for left APs (21.70% to 54.50%) were far from those expected, showing that none of the seven algorithms was significantly better than the others in predicting these APs correctly. However, if we include adjacent APs, the algorithm by D'Avila et al.^14^ (eight locations, 87% agreement rate) showed a value close to that expected and should be considered for locations close to left APs. In summary, the application of the seven algorithms to correctly predict septal, right, and left AP locations revealed that none of these algorithms achieved the expected results. However, the inclusion of adjacent APs allows the choice of a specific algorithm if the intention is to predict the approximate location of the AP. We conclude, therefore, that this choice is independent on the number of possible locations of each algorithm.

The agreement rates obtained with each algorithm are similar to those found by Wren et al.^9^ in a cohort of 100 children, which ranged from 29.50% to 48.50% when using the same algorithms. These authors also found that the algorithms by Arruda et al.^11^ and Chaing et al.^13^ had the highest corrected agreement rates for the number of possible anatomical locations (5.2 and 5.1 times more matches, respectively). The findings observed by these authors were similar to ours regarding the agreement rates and the number of possible locations by each algorithm (the absence of a relationship between these two parameters) and agreement among researchers (which was higher for algorithms identifying fewer locations).

The results obtained in the prediction of the precise location of the APs (match) were, as already noted, significantly lower than those published by the authors of each of the seven algorithms. This fact, common to the study of Wren et al.,^9^ was also observed in a study by Moraes et al.,^10^ in which the agreement rates obtained with the application of different algorithms in a sample of 190 patients with WPW syndrome proved to be substantially below those published by the authors of each algorithm (including, among others, the algorithm by Iturralde et al. with an agreement rate of 54.7%). Similar findings were obtained in a study published by Basiouny et al.,^23^ which compared 11 algorithms applied to 266 ECGs of patients with WPW syndrome. These researchers found significantly lower results for those algorithms with more than six possible locations for the AP. They also observed positive predictive values of 86% for APs with left lateral location, 45% for those with a right anteroseptal location, and 23% for those with a right posterolateral location.

All subjects included in our study presented a structurally normal heart. Bar-Cohen et al.^24^ analyzed children with WPW syndrome with and without congenital heart disease (43 children in each group, with a mean age of 5 and 15 years, respectively) using the algorithms described by Arruda et al.,^11^ Boersma et al.,^12^ and Fitzpatrick et al.^15^ These authors reported success rates ranging from 56-77% in children with structurally normal heart, which were significantly lower than those found in children with congenital heart disease (29-42%) when children with Ebstein's anomaly were excluded. Although these results were obtained in children and were lower than those expected, they are significantly higher than the ones obtained in our study, which included participants without structural heart disease.

The results obtained in our study reflect the limitations of the investigation and must be interpreted while keeping in mind that the analyzed algorithms were developed in adult patients, or in the case of the algorithm by Boersma et al.,^12^ in children. Thus, since the sample consisted of individuals aged between 7 and 75 years, the agreement rates obtained may have reflected important anatomical variations (such as the anatomical position of the heart relative to the chest) or differences in the ECG according to age. These results should also take into account a subjective interpretation of the ECG during application of the algorithms, for example, interpretation of the QRS complex polarity as positive or negative depending on the observer (the analysis of the agreement among the investigators seems to have demonstrated this issue).

The sequential use of each algorithm by the research team (leading to fatigue), the lack of knowledge or familiarity with the algorithms in clinical practice, as well as different levels of preexcitation in the analyzed ECGs and variations in the ECG technique may also explain the low accuracy rates obtained compared with those expected.

The actual location of a successfully performed AP ablation is the best parameter to identify the location of the AP. In contrast, location of the AP through ECG may be questionable, considering that APs may have a morphologically different ventricular insertion from the AP path. Thus, an ECG with a WPW pattern is dependent mainly on the location of the ventricular insertion of the AP and unrelated to its path. As described by Fox et al.,^25^ some algorithms tend to predict the APs correctly on a specific anatomic location, but may lead to error when the AP is located in other anatomic regions, such as a septal location. For these authors, the ECG, in fact, provides only an initial approach to the AP location.

## Conclusion

The ECG is an essential diagnostic method to identify a WPW ventricular preexcitation, but in our study, it had low sensitivity and specificity in locating the AP. Our analysis of seven algorithms revealed that none was able to achieve a high agreement rate.

The agreement rates for each algorithm did not increase with a decrease in precision, *i.e.* with fewer possible APs located by the algorithm. Regardless of the number of locations that each algorithm is able to identify, it was possible to highlight a single algorithm for each location (septal, right and left) as the most suitable to approximately estimate the location of the APs. It is noteworthy, however, that the agreement among the investigators was higher for algorithms identifying fewer AP locations.

The agreement rates obtained for each algorithm approached the results of other similar studies, which allows us to conclude that all analyzed algorithms have lower agreement rates than those published by their authors.
